# Hospital Meetings, &c.

**Published:** 1899-08-05

**Authors:** 


### HOSPITAL MEETINGS, &C.

### HOSPITAL SUNDAY FUND.

A meeting of the Council of the Metropolitan Hospital Sunday Fund was held on Wednesday, July 26th, at the Mansion House, to receive the report of the Committee of Distribution, and to order the payment of awards to hospitals and dispensaries. The Lord Mayor (the Right Hon. Sir John Voce Moore), president and treasurer, presided. Among those present were: The Sheriffs, Sir Sydney Waterlow, Bart. (vice-president), Archdeacon Sinclair, Canon Pennefather, Canon Rhodes Bristow, &c. Sir Savile Crossley, who was unable to be present, forwarded a cheque for £500.

The report of the Committee of Distribution presented to the meeting recommended awards for the year to 192 institutions, being four more than last year. The sum collected during the year, including ⅔ interest from the Court of Chancery, amounted to £50,716, and the committee state that they anticipate a further £2,000 before the accounts are closed on October 31st. The distribution of £50,500 to the 138 hospitals, &c., and to the 54 dispensaries, was advised. One hundred and ninety-five institutions had made applications for grants from the funds. Of this number the committee found it necessary to invite the attendance of deputations from the governing bodies of 18, and they were unable to recommend any awards in three cases. In addition to the £50,000 recommended for distribution, 5 per cent. of the total sum collected—viz., about £2,500—had been set apart to purchase surgical appliances in monthly proportions during the ensuing year. In conclusion the report stated that the amount available for distribution was larger than that distributed last year, owing to the munificent donation of £10,000 from Mr. George Herring. The collections on Hospital Sunday were generally larger than those of last year, showing that the confidence of the public in the Fund was well sustained.

The Lord Mayor, who was most cordially received, at the outset said it was a pleasant duty for him to have to welcome so distinguished a body of gentlemen to the Mansion House as he saw before him, assembled to promote the work of providing for the sick poor of London through the agency of hospitals by making awards to those institutions according to their merits and needs. He wished to express his gratitude to the public generally for their response to his appeal in the London newspapers, especially to raise this year the sum of £50,000, which had already been exceeded. (Cheers.) The Council had been most anxious to realise the total of £1,000,000 during the current year, and he congratulated them upon the fact that that sum had not only been reached, but somewhat exceeded. (Renewed cheers.) That was the sum which had been received since the establishment of the Fund in 1873. In conclusion he desired to thank most heartily Mr. George Herring for his munificent donation of £10,000, and Mr. Densham for his contribution of 1,000 guineas. (Cheers.) He called upon Sir Sydney Waterlow to propose the first resolution.

Sir Sydney Waterlow, who was cheered on rising, said it had been not only his privilege but his duty, as chairman of the Distribution Committee, ever since the Fund was established in 1873, to propose the first resolution, viz., "That the report of the Committee of Distribution for the year 1899 be and is hereby approved, and that the several awards recommended be paid as soon as possible." He did so with the greatest possible pleasure and satisfaction on this the twenty-seventh occasion. (Cheers.) All who were interested in the Fund would feel, he was sure, that, as it had been working under difficulties during the last three or four years, the large amount collected this year was evidence that the Fund still retained the confidence of the public and that it rested on a solid Christian basis—one which he believed would help them to make similar collections in years to come. The Lord Mayor had told them that they had during the period under review brought up the total amount received to £1,000,000, but as a matter of fact up to that moment the sum received, including the £500 so generously contributed by Sir Savile Crossley, had been £1,008,363—(cheers)—which was a large amount for his committee to have the responsibility of distributing. In addition to what he had stated the Fund had the interest on £45,000 of Consols left by the Guesdon trust and the reversion to £50,000 on the death of a lady. It was said, and with a great deal of truth, that the population of the metropolis increased so largely that a population almost equal to a reasonable sized provincial town was added to London every year. No doubt that was so, but let them look on the other side of the question: Had the public helped so far as they could to provide increased accommodation for the sick poor of the metropolis in proportion to the growth of its population? He thought that they had. Sir Sydney then proceeded to give the following facts to substantiate his assertion. When the committee began, in 1874, 17 general hospitals asked help from them, whereas the number this year was 31; the 6 convalescent homes had increased to 29; and the 3 cottage hospitals to 14. In 1874 only 1,100 collections in places of worship were taken by the Fund, but this year they had taken, or would take, collections from 1,900 places of worship. (Cheers.) The Council asked them that day to confirm the granting of awards amounting to £50,500, and, in addition, to approve the setting apart of £2,500 to purchasing surgical appliances —a total of £53,000. The members of the Council had always considered that they ought each year to distribute what they received, viz., that which they received in the year 1899 should provide for the sick poor of 1899, and trusting to the good feeling of the great community to provide in 1900 what was necessary for that year. Personally, he had always thought that a good plan, and he hoped it would be continued. (Hear, hear.) The church collections this year had been throughout larger than they were last year, and he welcomed this as the best possible sign they could have of the growing feeling as to the necessity of supporting the Fund. (Cheers.) The work of the committee had been greater during this year, and they anticipated it would increase in the coming twelve months. In conclusion he moved the resolution as above.

Archdeacon Sinclair seconded the motion, and the resolution was carried unanimously.

The Rev. G. R. Thornton moved: "That the cordial thanks of the Council be and are hereby given to Sir Sydney H. Waterlow, Bart. (chairman), and to the other members of the Committee of Distribution, for the care bestowed in the preparation of the awards they had recommended."

Mr. Thomas Bryant, in seconding the resolution, congratulated Sir Sydney Waterlow on having lived to see the fund, which he had been connected with from the first, attain the splendid success shown at the present time. (Cheers.)

Dr. Glover asked why the award to St. Thomas's Hospital was so small compared with the size and importance of the institution.

Sir Sydney Waterlow replied that the smallness of the amount awarded to St. Thomas's was solely on account of the very large endowment enjoyed by that hospital. To the London Hospital, on the other hand, they always gave the greatest sum, because its endowment was so very little. St. Bartholomew's, he mentioned, possessed an endowment so very large that it did not require any assistance.

Sir Edmund H. Currie proposed: "That the thanks of the Council be and are hereby given to the editors of newspapers who have pleaded the needs of hospitals and advocated the cause of this Fund."

Sir William Broadbent seconded the motion, which was carried unanimously.

Sir Dyce Duckworth moved, and the Rev. Canon Bristow seconded, the following resolution, which was agreed to *nem. con.*: "That the cordial thanks of the Council are now given to the Right Honourable Sir John Voce Moore, the Lord Mayor, who, as president and treasurer, has devoted his valuable time and influence to the well-being of the Fund."

The Lord Mayor having suitably replied, the proceedings terminated.

